# Rapidly progressive interstitial lung disease combined with pneumocystis jiroveci pneumonia in a patient with single anti-TIF-1γ antibody positive dermatomyositis in the context of an underlying tumor

**DOI:** 10.1186/s12890-023-02542-0

**Published:** 2023-07-06

**Authors:** Hengzhe Zhang, Jinfeng Yue, Xiaohui Hou, Hongjun Lu, Arezou Bikdeli, Haipeng Guo, Hao Li, Daqing Li

**Affiliations:** 1 Key Laboratory of Cardiovascular Remodeling and Function Research, Chinese Ministry of Education, Chinese National Health Commission and Chinese Academy of Medical Sciences, State and Shandong Province Joint Key Laboratory of Translational Cardiovascular Medicine, Department of Cardiology, Qilu Hospital, Cheeloo College of Medicine, Shandong University, Jinan, 250012 Shandong China; 2 Department of Intensive Care Medicine, Qilu Hospital, Shandong University, Jinan, 250012 Shandong China; 3 Department of Intensive Care Medicine, Traditional Chinese Medicine of Rizhao Hospital, Rizhao, 276800 Shandong China; 4 Department of Hematology, Qilu Hospital, Shandong University, Jinan, 250012 Shandong China

**Keywords:** Dermatomyositis, Pneumocystis jiroveci pneumonia, Interstitial lung disease, Rapidly progressive interstitial lung disease, Anti-transcriptional intermediate factors-1γ antibody, Rapid weight loss, Paraneoplastic syndrome, Clinical case

## Abstract

**Background:**

Interstitial lung disease (ILD) is a frequently observed comorbidity in autoimmune diseases such as dermatomyositis/polymyositis (DM/PM), and it is significantly associated with specific autoantibody types. One unique antibody type is the anti-transcription intermediate factor-1γ antibody (anti-TIF-1γ Ab), which has a positive rate of only 7%. It is often found in combination with malignancy and rarely with ILD, particularly rapidly progressive ILD (RPILD). In some cases, the presence of ILD in individuals with DM may indicate a paraneoplastic syndrome. Pneumocystis jiroveci pneumonia (PJP) typically occurs due to intensive immunosuppressive therapy, human immunodeficiency virus (HIV) infection, or malignancy, and rarely as an isolated condition.

**Case presentation:**

A 52-year-old man with a history of rapid weight loss but non-HIV infected and not immunosuppressed who presented with fever, cough, dyspnea, weakness of the extremities, characteristic rash and mechanic's hand. Pathogenic tests suggested PJP, laboratory tests suggested a single anti-TIF-1γ Ab positive DM, imaging suggested ILD, and pathology revealed no malignancy. RPILD and acute respiratory distress syndrome (ARDS) developed after anti-infection and steroid hormone therapy. After mechanical support therapy such as Extracorporeal Membrane Oxygenation (ECMO), the patient developed late-onset cytomegalovirus pneumonia (CMVP), complicated bacterial infection, and ultimately death. Additionally, we discuss the potential causes of rapid weight loss, the mechanisms by which anti-TIF-1γ Ab may lead to ILD, and the possible connection between anti-TIF-1γ Ab positivity, rapid weight loss, immune abnormalities, and opportunistic infections.

**Conclusions:**

This case emphasizes the importance of early recognition of malignant tumors and pulmonary lesions, assessment of the body's immune status, prompt initiation of immunosuppressive treatment, and prevention of opportunistic infections in individuals with single anti-TIF-1γ Ab positive DM presenting with rapid weight loss.

## Introduction

Dermatomyositis (DM) is a chronic autoimmune disease that commonly affects multiple organs, including the skin, transverse muscles, and lungs. The pathology of DM is characterized by tissue infiltration of inflammatory cells [1, 2]. Clinical practice has identified various myositis-specific autoantibodies (MSAs), such as anti-synthetic enzyme antibody and anti-melanoma differentiation associated gene 5 antibody (anti-MDA5 Ab). Among these, anti-histidyl tRNA synthetase antibody (anti-Jo-1 Ab) is the most prevalent, with a detection rate of approximately 18.7% in the European idiopathic inflammatory myopathies (IIM) population. On the other hand, the positive rate for anti-TIF-1γ Ab is only 7%, and the incidence of single positive anti-TIF-1γ Ab is as low as 6.4% [3]. It is noteworthy that anti-TIF-1γ Ab positive DM typically manifests with symptoms like dysphagia, cutaneous manifestations, or malignancies such as lung cancer, colon cancer, and non-Hodgkin's lymphoma, but it rarely leads to ILD [4, 5]. Long-term follow-up studies have demonstrated that patients with DM often have an increased risk of developing malignancies, with a standardized incidence ratio of approximately 5.50 (95% CI, 4.31–6.70). DM can also be diagnosed as a paraneoplastic syndrome before the detection of solid tumors, although it is less frequently associated with ILD (OR, 0.11; 95% CI, 0.02–0.51) [6, 7]. PJP is an opportunistic infection that typically occurs as a late complication in patients with HIV, malignancy, or long-term immunosuppression, often resulting in deterioration or even death [8, 9]. The occurrence of ILD in anti-TIF-1γ Ab positive DM/PM patients is rare, with an incidence of only 1.7% reported in a Japanese study, and the levels of this antibody significantly correlate with disease activity [10]. However, to the best of our knowledge, this is the first reported case of a single anti-TIF-1γ Ab positive DM patient with ILD as a potential manifestation of paraneoplastic syndrome in the presence of an underlying tumor context. Additionally, the development of PJP and RPILD in the absence of HIV infection and prior immunosuppression is remarkable. Despite receiving medication and mechanical support, the patient developed acute respiratory distress syndrome (ARDS) and respiratory failure (RF), and experienced late complications of CMVP and complicated bacterial infections, ultimately leading to death.

## Case presentation

The 52-year-old overweight male experienced a red rash with mild itching on the neck, back, and extensor surfaces of the elbow joints six months ago. The rash gradually faded, leaving behind dry skin, slight flaking, and hyperpigmentation by March 2022. However, in September 2022, he sought medical attention at the respiratory medicine department due to recurring symptoms. His symptoms included afternoon fever with a maximum temperature of 37.5°C, cough, sputum production, dyspnea, weakness, and limb aching. The patient did not have a history of using immunosuppressive drugs, and multiple tests came back negative for HIV. Notably, he had undergone significant weight loss of approximately 40 kg over the past 10 months through diet and exercise. In terms of his medical history, the patient had a 5-year history of hypertension and a 3-year history of drinking alcohol, though he was currently abstinent from alcohol consumption. His height was 177cm, weight was 82 kg, PaO_2_ (arterial blood oxygen partial pressure): 87 mmHg, PaCO_2_ (arterial blood carbon dioxide partial pressure): 30 mmHg, SO_2_ (saturation oxygen): 90%, lactic acid: 1.1 mmol/l. Clinical examination findings: temperature: 37°C, pulse: 84 beats/min, blood pressure: 131/81 mmHg, no obvious dysphagia, no joint pain, no neurological abnormalities. Skin findings: tiny red rashes under the neck and on the radial side of the fingers, rough skin on the tips and periphery of the fingers, and a few dark purple blebs around the nail bed. The results of the first admission laboratory tests are shown in Table 1(a).

**Table 1:** Laboratory blood text and autoimmune antibodies findings on admission (September 2022)

(a). Laboratory blood text items	Results	Reference values
WBC (× 10^9^/L)	8.03	3.5–9.5
**RBC (× 10**^**9**^**/L)**	**4.04**	4.3–5.8
**Hemoglobin(g/L)**	**120**	130–175
**Platelet (× 10**^**9**^**/L)**	**377**	125–350
**Neutrophil (× 10**^**9**^**/L)**	**7.53**	1.8–6.3
**Lymphocyte (× 10**^**9**^**/L)**	**0.42**	1.1–3.2
**C-reactive protein (mg/L)**	**25.19**	< 6
**Erythrocyte sedimentation rate (mm/h)**	**42**	0–15
**IL-6 (pg/mL)**	**146**	0–7
**(1,3)-β-D-glucan (pg/mL)**	**170.01**	70.00–95.00
Galactomannan	Negative	-
Cryptococcus podophyllum polysaccharide antigen	Negative	-
TB-ELISPOT	Negative	-
HIV	Negative	-
HBV, HCV	Negative	-
COVID-19	Negative	-
**IgE (IU/mL)**	**348**	< 100
**Carcinoembryonic antigen (ng/mL)**	**28.2**	0–5.0
**Cytokeratin 19 fragment (ng/mL)**	**9.9**	0–3.3
**Neuron specific enolase (ng/mL)**	**35.2**	0–16.3
ALT (U/L)	39	9–50
**AST (U/L)**	**53**	15–40
**Albumin (g/L)**	**24**	40.0–55.0
Globulin (g/L)	33	20.0–40.0
BUN (mmol/L)	5	2.3–7.8
**Cr (μmol/L)**	**46**	62–115
**LDH (U/L)**	**539**	120–230
CK (ng/mL)	38	38–174
CK-MB (ng/mL)	4	0.3–4.0
CD19 + B Lymphocyte (/μL)	209	107–698
**CD3 + T Lymphocyte (/μL)**	**340**	603–2990
**CD4 + T Lymphocyte (/μL)**	**233**	441–2156
**CD8 + T Lymphocyte (/μL)**	**110**	125–1312
**NK cell (μL)**	**34**	95–640
(b). Autoimmune antibodies		
Anti-SSA antibody	Negative	-
Anti-SSB antibody	Negative	-
Anti-Sm antibody	Negative	-
Anti-dsDNA antibody(IU/ml)	Negative	-
Anti-CCP antibody(RU/ml)	11.05	< 25
Rheumatoid factor(IU/ml)	< 30	< 30
PR3-ANCA(U/ml)	2.12	< 5
MPO-ANCA(U/ml)	2.06	< 5
ACL-IgG(U/ml)	7.39	< 10
ACL-IgM(U/ml)	1.56	< 10
Anti Jo-1 antibody	Negative	> 5 AU
Anti PL-7 antibody	Negative	> 5 AU
Anti PL-12 antibody	Negative	> 5 AU
Anti EJ antibody	Negative	> 5 AU
Anti SRP antibody	Negative	> 5 AU
Anti Mi-2 antibody	Negative	> 5 AU
Anti MDA-5 antibody	Negative	> 5 AU
**Anti TIF-1γ antibody**	**22**	> 5 AU
Anti SSA/Ro52 antibody	Negative	> 5 AU
Anti SAE-1 antibody	Negative	> 5 AU
Anti SAE-2 antibody	Negative	> 5 AU
Anti NXP-2 antibody	Negative	> 5 AU
Anti OJ antibody	Negative	> 5 AU
Anti KS antibody	Negative	> 5 AU
Anti ZO antibody	Negative	> 5 AU
Anti HA antibody	Negative	> 5 AU
Anti Scl-70 antibody	Negative	> 5 AU
Anti PM-Scl-100 antibody	Negative	> 5 AU
Anti PM-Scl-75 antibody	Negative	> 5 AU
Anti Ku antibody	Negative	> 5 AU
Anti RNA-P III antibody	Negative	> 5 AU
Anti Th-To antibody	Negative	> 5 AU
Anti Fibrillarin antibody	Negative	> 5 AU
Anti NOR-90 antibody	Negative	> 5 AU

**Fig. 1:**
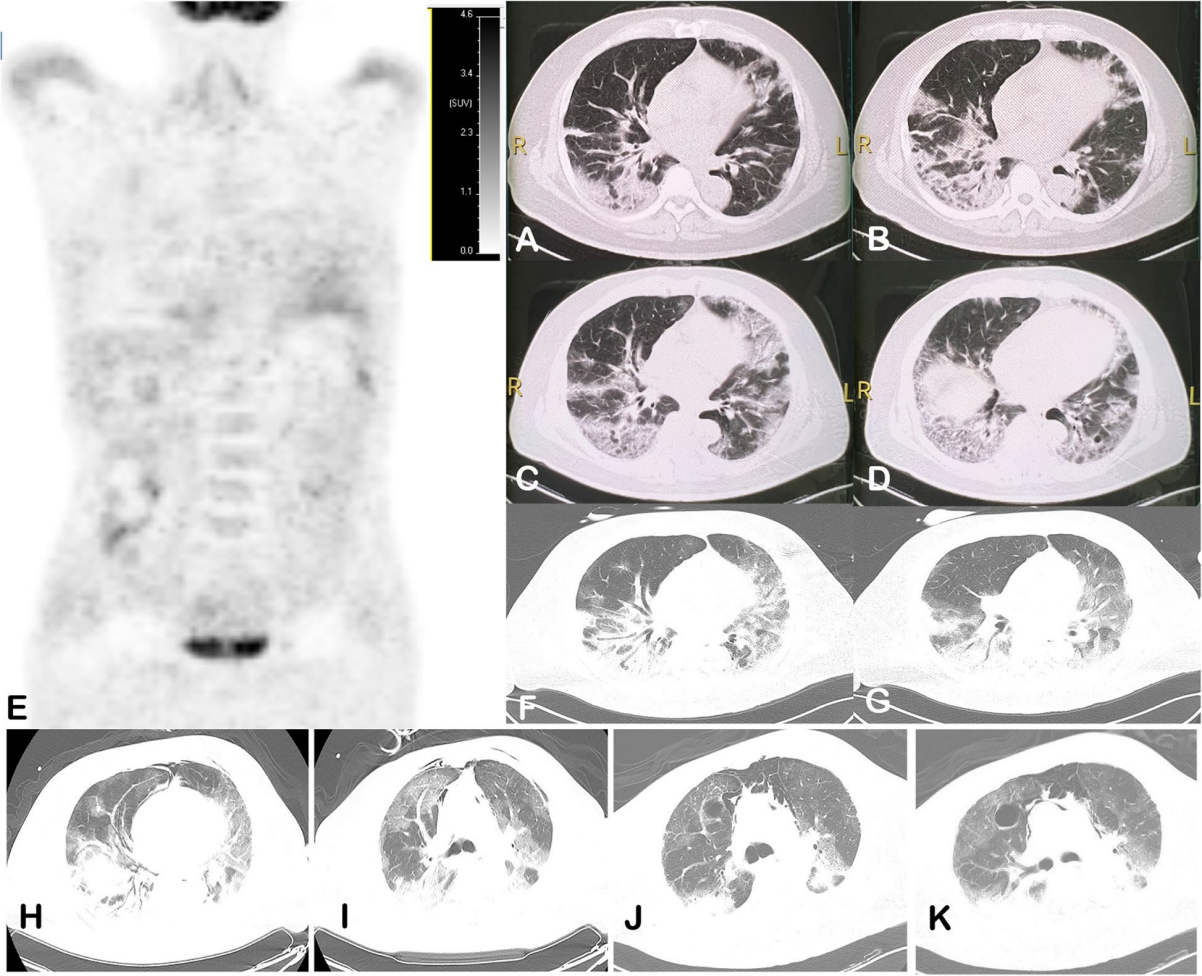
HRCT of the chest and whole body PET-CT fluorodeoxyglucose uptake Imaging. **A**, **B**: First visit to the previous hospital, CT showed ground glass opacities in both lobes of the lung. **C**, **D**: 1 week before treatment with cotrimoxazole, CT showed increased inflammatory exudate in both lungs with lattice-like and pavement-like lesions. **E**: Benignly increased FDG uptake in multiple muscles, no abnormal FDG uptake in tumour. **F**, **G**: Respiratory distress worse referral to ICU, CT showed inflammation in both lungs with some solidity and bronchial inflation signs. **H**, **I**: Complicated bacterial infections after intensive immunosuppressive therapy, CT showed no significant improvement in inflammation in both lungs, solid lung changes and significant interstitial inflammation. **J**, **K**: After upgrading antibiotic therapy, before evacuating ECMO, pathological finding was CMVP

The chest CT (Fig. 1 A, B) revealed that multiple ground glass density patchy and grid shadows in both lobes of the lung, with more severity in the right lung, which suggested pulmonary infection and interstitial lung lesions. Bronchoscopy revealed congestion of the bronchial mucosa. The next generation sequencing (NGS) of bronchoalveolar lavage solution (BLS) identified only nucleic acid sequences of Pneumocystis jirovecii (235kb), confirming the presence of Pneumocystis jirovecii colonization. After 7 days of treatment with Itraconazole and Piperacillin-tabazotam, there was no improvement in fever or respiratory symptoms. Subsequent chest CT (Fig. 1 C, D) revealed increased inflammatory exudate in both lungs, along with lattice-like and pavement-like lesions. Following treatment with oral and intravenous Cotrimoxazole, the patient's fever resolved, and respiratory symptoms improved. The effective response to anti-PJP treatment further supported the diagnosis of PJP. It's important to note that NGS is considered the gold standard for diagnosing Pneumocystis jirovecii colonization, and the combination of the patient's clinical symptoms, signs, imaging findings, and response to treatment all contribute to confirming the diagnosis of PJP [11].

**Fig. 2:**
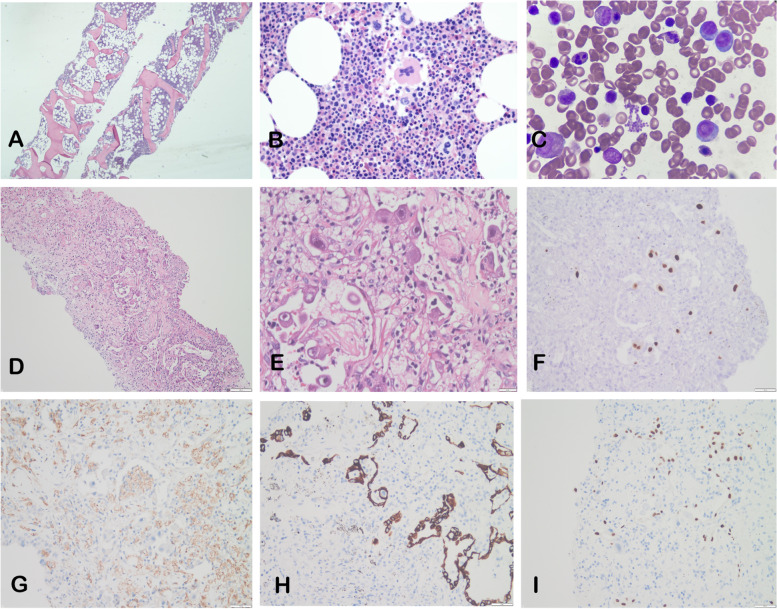
Bone marrow aspiration results (HE staining). **A** (4 × 10) & B (10 × 10): No significant histopathological abnormalities in the bone marrow; **C**: No anisocytic cells on bone marrow smear. Pathological findings of CT guided right lung tissue puncture biopsy. HE staining, **D**(10 × 10) & **E**(40 × 10): Chronic inflammation of lung tissue atypical alveolar epithelial hyperplasia and interstitial oedema, foam cell hyperplasia and focal necrosis in the alveolar lumen, and viral inclusion bodies. IHC staining: **F** (CMV +), **G** (CD68 -), **H** (CK7 +), **I** (TTF-1 +)

The results of the MSAs assay indicated that only anti-TIF-1γ Ab IgG was positive (Test method: Line blot assays; Sensitivity: 40%; Specificity: 96%; Anti-TIF-1γ Ab content: 22 AU), as shown in Table 1(b) [12]. Muscle and skin biopsies were not performed due to a probability of over 90% of DM based on the International Myositis Classification Criteria Project (IMCCP) (age > 50 years: 2.1; characteristic rash and mechanic's hand: 2.1; Anti-TIF-1γ Ab positive, as well as elevated aspartate aminotransferase and lactate dehydrogenase: 5.2; overall score: 9.4 > 7.5, corresponding to a probability of 90%) [13]. To rule out the possibility of a tumor, a PET-CT scan revealed interstitial changes in both lungs, emphysema, benign high fluorodeoxyglucose (FDG) uptake in multiple muscle tissues, and no tumor FDG high uptake was detected (Fig. 1E, SUV maximum for lung tissue: 2.67, mean: 0.74; SUV maximum for upper limb muscle tissue: 2.58, mean: 1.05). An SUVmax > 2.4 for lung FDG uptake has been reported as a sensitive indicator for the diagnosis of RPILD [14]. Additionally, no tumor was found on bone marrow aspiration cytology and pathology (Fig. 2 A-C). Therefore, the patient was treated with intravenous steroids and human immunoglobulin while continuing anti-infective treatment. After 3 days of treatment, the patient suddenly developed respiratory distress, became unconscious and entered a coma. Blood gas analysis revealed PaCO_2_: 37 mmHg, PaO_2_: 44 mmHg, Lac: 3.4 mmol/l and SO_2_: 60%. Ultrasound examination showed the presence of massive B lines in the lungs.

Consequently, the patient was transferred to the intensive care unit for further treatment. A chest CT scan (Fig. 1 F, G) revealed inflammation in both lungs with partial solidity and signs of bronchial inflation. Considering the patient's symptoms and investigative findings, DM related RPILD, ARDS, and RF were suspected. Tracheotomy intubation, low tidal volume (6 ml/kg), continuous positive end-expiratory pressure invasive ventilation, VV-ECMO, and continuous renal replacement therapy were administered. After 1 week, bronchoscopy was repeated, and NGS testing for Pneumocystis jirovecii did not reveal any presence of the pathogen. Serum (1,3)-β-D-glucan and galactomannan tests were negative. Intensive immunosuppressive therapy was initiated based on the recommendation of the rheumatologist, including the administration of methylprednisolone and cyclophosphamide. After 2 weeks of treatment, the patient began producing a large amount of yellowish-white mucous sputum. Bronchoscopy revealed significant retention of viscous yellowish-white sputum in both lungs. Aspiration therapy was performed, and sputum culture and drug sensitivity testing were conducted, which indicated a mixed infection of Klebsiella pneumoniae and Acinetobacter baumannii.

Subsequently, a chest CT scan (Fig. 1 H, I) showed no significant improvement in lung inflammation compared to previous scans, with persistent solid lung changes and significant interstitial inflammation. Consequently, the antibiotic regimen was upgraded to Imipenem and Polymyxin E. Despite several attempts to wean the patient off ECMO, adequate oxygen saturation and blood pressure could not be maintained. A follow-up chest CT scan (Fig. 1 J, K) revealed multiple areas of interstitial inflammation in both lungs, along with pulmonary solidity and atelectasis. Informed consent from the family was obtained, and ECMO support was discontinued. A CT-guided puncture biopsy of the lung tissue was performed, revealing pathological findings indicative of chronic inflammation and interstitial edema. Mild atypical hyperplasia of the alveolar epithelium and foam cell hyperplasia in the alveolar lumen were also observed. Focal necrosis with detectable viral inclusion bodies (Fig. 2 D, E) was present, and immunohistochemistry suggested CMV infection (Fig. 2 F-I). Based on these findings, CMVP was considered as a consequence of immunosuppressive therapy. Unfortunately, due to the high medical costs and extremely poor prognosis, the family decided to discontinue further treatment. The clinical course of the patient is summarized in Table 2.

**Table 2:** Timeline of the disease and treatment

Timeline	Symptoms/Texts	Dignosis	Treatment	Outcome
March 2022	Gottron’s papules, V neck syndrom, weakness and soreness of limbs, ect	None	None	The rash fades and leaves dry skin, a little flaking and hyperpigmentation
September 2022	Fever, cough, sputum, dyspnea, Gottron’s papules, V neck syndrom, weakness and soreness of limbs, weight lose; CT: exudative lesions and interstitial lesions in both lungs; NGS: Pneumocystis jirovecii; (1,3)-β-D-glucan ( +); IgE significant increase; HIV(-)	Non-HIV PJP	Itraconazole (0.2 g/2 times daily); Piperacillin tabazotam (4.5 g/3 times daily); Cotrimoxazole (oral: 1.6 g/4 times daily; ivdrip: 0.8 g/3 times daily)	No improvement in fever and respiratory symptoms
7 October 2022	Gottron’s papules, V neck syndrom, Mechanic's hand, significant increase in LDH & AST,anti-TIF-1γ Ab( +), IMCCP score > 7.5; PET-CT:interstitial changes in both lungs, no high FDG uptake of tumour detected; No tumour found on bone marrow aspiration cytology and pathology	Non-HIV PJP; DM combined with ILD	Piperacillin tabazotam (4.5 g/3 times daily); Cotrimoxazole (oral: 1.6 g/4 times daily; ivdrip: 0.8 g/3 times daily); Methylprednisolone (500 mg/2 times daily); Human immunoglobulin (2.5 g/1 time daily)	Return of normal body temperature, improvement in respiratory symptoms and resolution of rash
10 October 2022	Increased dyspnea, unconsciousness, coma; Blood gas analysis:PaCO_2_ 37 mmHg, PaO_2_ 44 mmHg,Lac 3.4 mmol/l, SO_2_ 60%; CT:inflammation of both lungs, partially solid with bronchial inflation signs, interstitial inflammation of lungs; Ultrasound: massive B lines present in the lungs; NGS (-); (1,3)-β-D-glucan and galactomannan (-)	DM combined with RPILD; ARDS; RF	Piperacillin tabazotam (4.5 g/3 times daily); Cotrimoxazole (oral: 1.6 g/4 times daily; ivdrip: 0.8 g/3 times daily); Methylprednisolone (400 mg/2 times daily); Human immunoglobulin (2.5 g/1 time daily); Cyclophosphamide (0.6 g/1 time per week); Invasive ventilation; VV-ECMO; Continuous renal replacement	Rash resolving, requiring mechanical maintenance therapy
31 October 2022	Massive yellow-white mucous sputum; Bronchoscopy: massive viscous yellow-white sputum retention in both lungs; CT:no significant improvement in inflammation in both lungs compared to before, with solid lung changes and marked interstitial inflammation; Sputum culture:mixed Klebsiella pneumoniae and Acinetobacter baumannii infection; Lung tissue biopsy: CMV infection	DM combined with RPILD; ARDS; RF; CMVP; Bacterial pneumonia	Imipenem cilastatin(500 mg/4 times daily); Polymyxin E(150 mg/2 times daily); Invasive ventilation; VV-ECMO; Continuous renal replacement; Aspiration therapy	Death after evacuation for mechanical support

## Discussion

ILD is a relatively common pulmonary manifestation among the extra muscular symptoms of DM. Studies have indicated that patients with positive anti-synthetase antibodies are more likely to develop ILD compared to those with other myositis-specific autoantibodies. In some cases, ILD may even be the initial and sole presenting symptom of DM, with an incidence ranging from approximately 71% to 100%. HRCT findings in DM-ILD commonly demonstrate patterns of non-specific interstitial pneumonia and organizing pneumonia, and most DM patients with concurrent ILD tend to respond well to steroid hormone therapy [15, 16]. Here, we present an unusual case of a single anti-TIF-1γ Ab positive DM patient, with a history of rapid weight loss but no HIV and immunosuppressant use, who developed PJP and ILD. Despite the administration of adequate anti-infective, immunosuppressive and mechanical supportive therapy, RPILD and ARDS developed and eventually death. This case serves as an important reference point for exploring potential mechanisms involving anti-TIF-1γ Ab and DM-ILD, autoimmune antibodies, malnutrition and the development of PJP in an underlying tumor context.

In this particular case, the weight loss was attributed to a long-term history of dietary restriction and exercise, which could be clearly traced. It is worth noting that autoimmune diseases can also be significant contributors to weight loss, and studies have demonstrated that weight loss is a common early symptom of IIM and is strongly associated with disease severity [17]. Although PET-CT and pathology findings do not support a solid tumor, the elevated tumor markers and positive anti-TIF-1γ Ab mean that we still cannot completely rule out the possibility of rapid weight loss due to an underlying malignancy.

Both PJP and autoimmune diseases are recognized as important causes of ILD [18, 19]. The connection between PJP and ILD can be approached from two possibilities: PJP may occur prior to ILD and directly contribute to its development, or PJP may occur subsequent to ILD, exacerbating DM-ILD and leading to RPILD. In the present case, despite receiving standard anti-PJP treatment, the patient's interstitial lung lesions persisted, and no further detection of Pneumocystis jirovecii nucleic acid sequences was observed through NGS. Therefore, it was concluded that ILD in this case was primarily associated with DM. The global prevalence of ILD in DM/PM has been reported to be approximately 41%, with a higher incidence observed among Asian populations [20]. Retrospective studies from Asia have demonstrated that the probability of DM combined with ILD as a manifestation of paraneoplastic syndrome in the context of solid tumors was approximately 4.16% (1/24), while the probability of clinically amyopathic DM (CADM) combined with ILD and malignancy was around 6.67% (1/15). European studies have also indicated that the incidence of DM/PM combined with ILD and malignancy was 5.71% (4/70), although multiple regression analysis showed that the presence of combined ILD reduced the likelihood of malignancy [21, 22]. Furthermore, specific studies focusing on anti-TIF-1γ antibody-positive cases have shown varying rates of ILD occurrence. For instance, Bodoki L et al. reported pulmonary fibrosis in 16.7% of 12 anti-TIF-1γ Ab positive patients out of a cohort of 337 Hungarian patients with IIM. Ikeda N et al. reported ILD in 22.6% (7/31) of anti-TIF-1γ Ab positive DM patients. Shimizu K. et al. described combined ILD in 8.69% (2/23) of anti-TIF-1γ Ab positive DM/CADM patients [23–25]. Considering the single anti-TIF-1γ Ab positivity in the presented case, we can speculate on potential mechanisms underlying its occurrence.

**Fig. 3:**
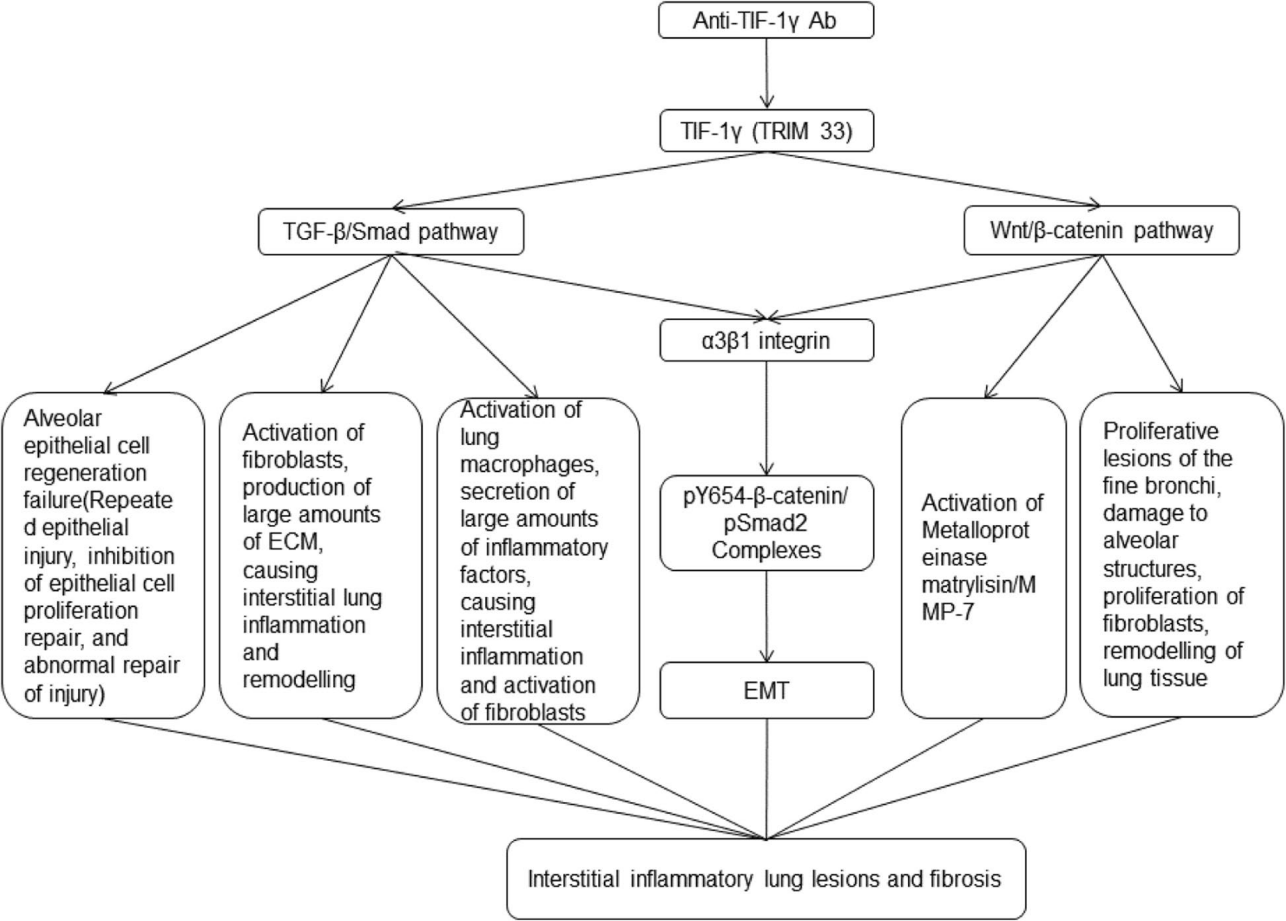
Anti-TIF-1γ Ab may deplete endogenous TIF-1γ (TIRM33) and attenuate the inhibition of the TGF-β/Smad pathway and the Wnt/β-catenin pathway. Excessive activation of these two pathways, in addition to causing tumors, can also cause interstitial inflammatory lesions, fibrosis and tissue remodelling in the lung via lung epithelial cells, fibroblasts and lung macrophages; activation of Activation of Metalloproteinase matrylisin/MMP-7, or, with the assistance of a3β1 integrin, the formation of pY654-β-catenin/ pSmad2 Complexes, leading to EMT, causing interstitial lung lesions and fibrosis. ECM: Extracellular matrix; EMT: Epithelial-mesenchymal transition; pY654: phosphorylation at tyrosine residue 654

Tripartite motif (TRIM) proteins are a family of ubiquitin ligases that modify proteins. Among the four isoforms of TRIM, TRIM33 (also known as TIF-1γ) is one of them. TIF-1γ plays a role in inhibiting the formation of Smad2/3/4 complexes and downregulating the TGF-β/Smad pathway. It achieves this by ubiquitinating Smad4 or acting as a cofactor for the phosphorylated Smad2/3 complex, thereby inhibiting epithelial-mesenchymal transition (EMT) [26, 27]. Additionally, TIF-1γ is involved in the Wnt/β-catenin pathway, where it promotes the degradation of β-catenin through ubiquitination, thereby influencing cell proliferation [28]. Studies have demonstrated that TGF-β is highly expressed in lung tissue affected by ILD, particularly in alveolar epithelial cells and lung macrophages. TGF-β and its downstream effectors inhibit the proliferative repair of epithelial cells in response to inflammatory injury and promote the overactivation of EMT, fibroblasts, and myofibroblasts, contributing to lung fibrosis [29]. Conversely, depletion of TRIM33 leads to increased production of TGF-β1, inducing idiopathic pulmonary fibrosis (IPF) and interstitial lung inflammation through Smad4 and downstream inflammatory factors such as TNF-α and IL-6 [30]. In IPF, the Wnt protein is overexpressed in alveolar epithelial cells and stimulates collagen secretion by fibroblasts [31]. Furthermore, the Wnt/β-catenin pathway is overactivated in fibroblasts during interstitial lung inflammation, and it exhibits an epithelial integrin-dependent profibrotic effect along with Smad signaling [32]. The mechanism by which anti-TIF-1γ Ab affects intracellular TRIM33 function is still under investigation. Some studies propose that intracellular TIF-1γ acts as an autoantigen and is immunogenic enough to elicit an immune response in the organism, leading to the production of anti-TIF-1γ Ab that affect intracellular metabolism and antibody efflux. Other studies suggest that when DM occurs, large amounts of TIF-1γ in the skin and muscle produce significant quantities of antibodies that impact intracellular metabolism through interactions with EMT, β-linked proteins, and mitotic processes [5, 33, 34]. In Fig. 3, we present a speculative model where the presence of a single positive anti-TIF-1γ Ab depletes TIF-1γ, potentially mediating interstitial pneumonia by upregulating the TGF-β/Smad and Wnt/β-catenin pathways. However, further studies are needed to confirm this hypothesis.

In DM, PJP is typically observed in patients undergoing long-term immunosuppressive therapy, especially in combination with ILD, HIV or malignancy [35]. However, in this case, the patient was HIV negative and had not received prior immunosuppressive therapy. Multiple lymphocyte type tests consistently reported low T and NK cell counts, indicating a state of cellular immunosuppression that was not primarily associated with later immunosuppressive therapy. Peripheral blood flow cytometry typing confirmed a significant reduction in total T cells in DM patients positive for anti-TIF-1γ Ab compared to other myositis antibodies. Additionally, TIF-1γ can regulate NKT cell differentiation through the TGF-β pathway and influence immune status [36, 37]. The patient's serum albumin levels remained low, which may be attributed to dietary restriction as well as disease-related depletion. However, a comprehensive assessment of the patient's nutritional status, including vitamins, amino acids and trace elements was not conducted. Studies have demonstrated that dieting for more than 6 weeks leads to a significant decrease in serum albumin levels. Both human and animal models have shown that hypoproteinemia weakens the body's immunity, reduces the number and activity of immune cells, and increases the susceptibility to infections and mortality [38, 39]. Moreover, rapid weight loss can result in a significant decrease in peripheral blood NK cell numbers and activity in obese women, thereby suppressing the immune response [40]. Paraneoplastic syndrome can also cause immune disorders, but we have not found definitive evidence that paraneoplastic syndrome causes a decrease in T lymphocytes and NK cells in human peripheral blood. Paraneoplastic syndrome can also cause immune disorders, but definitive evidence linking paraneoplastic syndrome to decreased T lymphocytes and NK cells in human peripheral blood is lacking. In this case, it is speculated that DM, dietary restriction, rapid weight loss, and malnutrition leading to an abnormal immune state are significant factors contributing to opportunistic infections.

In this case, the chest CT findings, including grid-like and pavement-like changes, acute exacerbation of exudative ground glass-like patchy shadow, and pulmonary solidity, are consistent with the presentation of RPILD [41]. Ultrasound examination revealed the presence of massive B lines in the lungs, and blood gas analysis indicated poor oxygenation, requiring invasive ventilation and VV-ECMO support therapy, which is consistent with ARDS and RF. Although the possibility of an underlying malignancy and paraneoplastic syndrome cannot be completely ruled out, due to the lack of permission for autopsy and the inability to follow up with the patient, a surgical lung tissue biopsy to confirm the ILD was not performed.

## Conclusions

This case report provides further evidence for the association of anti-TIF-1γ Ab positive DM with ILD as a possible manifestation of paraneoplastic syndrome in the context of an underlying tumor. The presence of anti-TIF-1γ Ab, along with factors such as rapid weight loss and malnutrition, may contribute to immune suppression and increase the risk of opportunistic infections, including RPILD, ultimately leading to death. In cases of anti-TIF-1γ Ab positive DM, it is important to consider malignancy as the primary comorbidity. Lung imaging evaluation is particularly crucial, especially in patients with opportunistic infections, as the development of RPILD should be closely monitored. Early detection of tumors, timely initiation of immunosuppressive therapy, improvement of immune status and infection prevention are important considerations in similar cases. Further research is needed to better understand the underlying mechanisms of this association and to explore effective clinical treatment options for patients with anti-TIF-1γ Ab positive DM and ILD in the context of malignancy.

## Data Availability

All data and materials supporting our findings are contained within this published article.

## Abbreviations

ILD: Interstitial lung disease
DM: Dermatomyositis
PM: Polymyositis
RPILD: Rapidly progressive interstitial lung disease
PJP: Pneumocystis jiroveci pneumonia
HIV: Human immunodeficiency virus
ECMO: Extracorporeal membrane oxygenation
CMVP: Cytomegalovirus pneumonia
ARDS: Acute respiratory distress syndrome
MSAs: Myositis-specific autoantibodies
IIM: Idiopathic inflammatory myopathies
RF: Respiratory failure
NGS: Next generation sequencing
BLS: Bronchoalveolar lavage solution
PaO_2_: Arterial blood oxygen partial pressure
PaCO_2_: Arterial blood carbon dioxide partial pressure
SO_2_: Saturation oxygen
CADM: Clinically amyopathic DM
TRIM: Tripartite motif
TIF: Transcription intermediate factor
MDA5: Melanoma differentiation associated gene 5
EMT: Epithelial-mesenchymal transition
IPF: Idiopathic pulmonary fibrosis
PET-CT: Positron emission tomography computed tomography
HRCT: High resolution computed tomography
